# London School of Tropical Medicine

**Published:** 1899-05-06

**Authors:** 


### The Hospital

A Journal of

The Medical Sciences and Hospital Administration.

Vol. XXVI.—No. 658.] Edited by Sir Henry Burdett, K.C.B., and Solomon C. Smith, M.D., M.R.C.P. [May 6, 1899.

### London School of Tropical Medicine.

This school, which owes its origin to Mr. Chamberlain, Secretary of State for the Colonies, has commended itself to public opinion, as is shown by the long list of influential people who have already contributed to the building and endowment funds. Mr. Chamberlain, at the outset, addressed a letter to each of the medical schools of London, and also to the authorities of the Seamen's Hospital at Greenwich, in order to ascertain which, if any, of them would co-operate with the Colonial Office in establishing a school with the object of affording instruction in tropical medicine to medical officers in the colonial service. None of the institutions addressed saw their way to affording the necessary facilities, with the exception of the "Dreadnought" Seamen's Hospital. In view of this fact, it is not a little surprising to realise that, when it became known that a school of tropical medicine would be established at the Branch Hospital at the Royal Victoria and Albert Docks, such school being replete with every requisite for adequate teaching and instruction, certain teachers in the London schools should have written to the papers deprecating the project, and urging that it would damage existing schools, without effecting any adequate purpose. We have no intention of pursuing this side issue, nor the further and most regrettable one which culminated in the resignation of certain members of the visiting medical staff of the "Dreadnought" Hospital. Personal questions of this kind have little or no public interest, and the result has proved once more that those who are responsible for raising such issues surely end in damaging no one but themselves.

The hospital to which this school will be attached is so situated as to secure the admission to its wards of a large number of Europeans of all nations, in addition to Asiatics, Africans, West Indians, and natives of British and other colonies, suffering from acute and chronic diseases commonly met with in actual practice in the tropics. The hospital contains forty-five beds, in addition to some two hundred which are provided by the Seamen's Hospital Society in the main institution at Greenwich. There is little doubt, therefore, that the material for clinical instruction will be abundant, and that the knowledge and experience gained in this school will conduce not only to the lessening of suffering and the preservation of life, but to the general welfare and health of those Englishmen who reside in the tropics. In addition to the clinical material, it will of course be essential that all modern teaching, appliances, including a bacteriological laboratory, a pathological department, model lecture room accommodation, and every modern appliance of a fully equipped medical school, should be provided. Mr. Chamberlain was careful to insist upon such provision being made, and the committee of the Seamen's Hospital are to be congratulated upon the thoroughness with which they have entered into the subject. All the cadets of the Colonial medical service will pass through the school, and the institution will also be open to all Government medical officers, as well as to medical men and students desirous of availing themselves of the opportunities which will be provided for studying tropical diseases. Those who enter the school will be required to attend a systematic course of instruction by the medical superintendent, in addition to the clinical lectures and demonstrations of the visiting medical and surgical staff. The scale of fees is moderate, and Mr. Chamberlain, with great practical common-sense, has secured adequate accommodation for resident students within the school buildings. The syllabus of the school, which has just been issued, shows that in arranging the courses of lectures care has been taken to include the study of every tropical disease, in addition to lectures on the "Hygiene of the Tropics, and the Sanitary Measures Necessary to Check Epidemics."

Great praise is due to Mr. Chamberlain for the personal interest he has shown in this school, and to him is due the chief credit for its successful establishment. He has consented to preside at a dinner which will be held on Wednesday next, the 10th inst., when we hope that he may have the satisfaction of announcing that the whole of the necessary money has been provided by the public. The total cost of the scheme is upwards of £16,000, and, besides this, it will be necessary to provide an income of £3,000 per annum. Altogether, including the contributions of the Colonial and Indian Offices, the sum already raised exceeds £10,000, in addition to a revenue of some £1,500, representing half the actual income required. When we remember how many and important are the commercial interests which depend upon the trade with the Tropics for their success, we cannot doubt that Mr. Chamberlain will be placed in the position of announcing at the dinner next week that the £6,000 still required has been subscribed, and that the finances of the school have been placed once and for all upon a sound and permanent basis. The Seamen's Hospital Society in entering upon this undertaking have shown much public spirit, and deserve the thanks of everybody who takes an intelligent interest in the growth and development of the British Empire. It is no small matter that the Seamen's Hospital should have courageously undertaken to afford the necessary facilities for the education of medical men who may be called upon to treat our own countrymen in the Tropics, where they will be enabled by the knowledge acquired at this school to immeasurably add to the reputation of England, and to successfully remove many of the causes which have heretofore rendered life in the Tropics almost an impossibility for Europeans. The teaching of medical science and the spread of civilisation have proved beyond dispute that it is often possible to render the most unhealthy climates reasonably safe for Europeans by the introduction of sound hygienic conditions and the continuous enforcement of precautions which have their origin in knowledge, and are applied with zealous devotion to the interests of the community.

